# Higher Anti-Drug Antibody Levels to Anti-Tumor Necrosis Factor Therapies Are Associated with Treatment Failure in Patients with Inflammatory Bowel Disease

**DOI:** 10.3390/jcm15020547

**Published:** 2026-01-09

**Authors:** Alessandra Saraga, Tina Deyhim, Ajay Gade, Grace Geeganage, Mostafa Soliman, Nathan David Vanshelboym Rothschild, Samantha Zullow, Loren G. Rabinowitz, Laurie B. Grossberg, Adam S. Cheifetz, Konstantinos Papamichael

**Affiliations:** Division of Gastroenterology and Hepatology, Beth Israel Deaconess Medical Center, Harvard Medical School, Boston, MA 02115, USA; asaraga@bidmc.harvard.edu (A.S.); tina.deyhim@lahey.org (T.D.); agade1@bidmc.harvard.edu (A.G.); msolima5@bidmc.harvard.edu (M.S.); nrothsch@bidmc.harvard.edu (N.D.V.R.); szullow@bidmc.harvard.edu (S.Z.); lrabinow@bidmc.harvard.edu (L.G.R.); lgrossbe@bidmc.harvard.edu (L.B.G.); acheifet@bidmc.harvard.edu (A.S.C.)

**Keywords:** Crohn’s disease, ulcerative colitis, inflammatory bowel disease, infliximab, therapeutic drug monitoring, adalimumab, anti-drug antibodies, immunogenicity

## Abstract

**Background/Objectives:** There is limited data regarding the association of anti-drug antibody (ADA) levels with the efficacy of anti-tumor necrosis factor (anti-TNF) therapy in patients with inflammatory bowel disease (IBD). We aimed to investigate the association between antibody to adalimumab (ATA) and antibody to infliximab (ATI) levels and treatment failure in IBD. **Methods:** This single-center, retrospective cohort study included consecutive IBD patients with ADA evaluated with a drug-tolerant assay between September 2012 and February 2023. A time-to-event analysis was performed for treatment failure, defined as the need for drug discontinuation due to primary non-response, loss of response, a serious adverse event, or an IBD-related surgery. Patients were followed from first positive ADA until treatment failure or the end of the follow-up (May 2024). **Results:** The study population consisted of 134 patients with IBD [n = 58 (43%) on adalimumab; n = 86, (64%) with Crohn’s disease]. Multiple COX regression analysis identified higher ADA levels to be associated with treatment failure (HR: 1.034, 95%CI: 1.024–1.045, *p* < 0.001). A ROC analysis identified an ATA and ATI level threshold of 5.2 U/mL (AUC: 0.705; 95%CI: 0.569–0.841; *p* = 0.003; sensitivity: 64%; specificity: 82%) and 8.8 U/mL (AUC: 0.809; 95%CI: 0.713–0.906; *p* < 0.001; sensitivity: 69%; specificity: 93%), respectively, to distinguish patients with or without treatment failure. **Conclusions:** In this large retrospective cohort study, higher levels of ADA were associated with treatment failure to anti-TNF therapy in IBD. Moreover, we identified ATA and ATI level thresholds of 5.2 U/mL and 8.8 U/mL, respectively, to be associated with treatment failure.

## 1. Introduction

Anti-tumor necrosis factor (anti-TNF) therapies, such as infliximab (IFX) and adalimumab (ADM), are foundational in managing moderate-to-severe inflammatory bowel disease (IBD), ulcerative colitis (UC), and Crohn’s disease (CD) [1]. However, their effectiveness is not universal. Up to 30% of patients do not respond and are considered as primary non-responders, while up to 50% after an initial response lose response over time [2]. Therapeutic drug monitoring (TDM) has revealed that a key contributor to these unfavorable outcomes is immunogenicity, defined as the formation of anti-drug antibodies (ADA) [2].

Immunogenicity is a major challenge of anti-TNF therapies leading to increased drug clearance and undetectable or low drug concentrations [3,4]. This is reflected by worse clinical outcomes including drug discontinuation and increased risk of infusion reactions [5,6,7,8,9,10,11]. One meta-analysis of 68 studies showed that antibodies to infliximab (ATI) are associated with lower clinical response rates and higher rates of infusion reactions [8]. Moreover, current data suggest that higher ADA are associated with worse outcomes [12,13,14,15,16]. For example, a retrospective cohort study demonstrated that patients with ATI levels higher than 8.8 U/mL had greater drug discontinuation [15].

However, there is misperception regarding the cut-offs utilized for the definition of high vs. low ADA levels and the thresholds associated with negative clinical outcomes in IBD. Additionally, various assays use different units to measure ADA, such as μg/mL, ng/mL or U/mL (i.e., the enzyme-linked immunosorbent assay vs. the homogeneous mobility shift assay (HMSA)) [17]. Another confounding factor is whether the TDM assay is drug-sensitive or drug-tolerant [18]. Drug-sensitive assays can measure ADA only when drug concentrations are undetectable, while drug-tolerant assays can measure ADA even in the presence of drug concentrations [18].

Preliminary data regarding the widely used drug-tolerant HMSA suggest that ATI levels higher than a threshold of 8.5 to 10.0 U/mL are associated with worse outcomes and are difficult to overcome, while the cut-off for antibodies to adalimumab (ATA) associated with negative outcomes remains largely unknown [17]. Other knowledge gaps regarding the impact of ADA on the efficacy of anti-TNF therapy include the presence or absence of drug concentrations and the role of reactive compared to proactive TDM when first detecting ADA.

This study aimed to investigate the association of ADA levels with treatment failure and to identify ATA or ATI level thresholds distinguishing patients with or without treatment failure. Secondary outcomes were to determine whether it was relevant if drug concentration was detectable or undetectable at the time of ADA and to determine if outcomes were different when ADA were identified by reactive vs. proactive TDM.

## 2. Methods

### 2.1. Study Design, Population, and Outcomes

This retrospective, single-center cohort study included consecutive IBD patients with ATA or ATI evaluated with a drug-tolerant assay from September 2012 to February 2023. Treatment failure was defined as the need for drug discontinuation due to primary non-response, loss of response, a serious adverse event (SAE) or surgery. Treatment modifications after the detection of ADA were based on the discretion of the treating physician and included dose increase, shortening of the dosing interval, addition of an immunomodulator (thiopurines or methotrexate), combination of the above, or drug discontinuation reflecting real-life clinical practice. Approval was obtained from the institutional review board at Beth Israel Deaconess Medical Center, Boston, MA, USA.

### 2.2. Therapeutic Drug Monitoring

Proactive TDM was defined as the evaluation of drug concentrations and ADA in patients with clinical remission or response based on symptoms and physician global assessment, while reactive TDM was defined as the assessment of drug concentrations and ADA in patients with a suspected loss of response or a SAE. Both proactive and reactive TDM were performed with the aim of achieving and maintaining drug concentrations typically higher than 5–10 μg/mL for infliximab and 10–15 μg/m for adalimumab. Serum adalimumab and infliximab concentration as well as ATA and ATI were measured using the homogeneous mobility shift assay (HMSA) (Prometheus Laboratories Inc., San Diego, CA, USA) [19,20]. The lower limit of quantification (LLOQ) for infliximab concentration was 1 μg/mL, while the LLOQ for ATI was 3.1 U/mL [19]. The LLOQ for adalimumab concentration was 1.6 μg/mL, while the LLOQ for ATA was 1.7 U/mL [20]. The upper limit of quantification (ULOQ) for infliximab concentration was 34 μg/mL, while the ULOQ for ATI was 100 U/mL [19]. The ULOQ for adalimumab concentration was 50 μg/mL, while the ULOQ for ATA was 55 U/mL [20]. Anti-drug antibody levels and drug concentrations < LLOQ were treated as “0,” whereas values higher than the ULOQ were treated as ULOQ + 1 [21].

### 2.3. Statistical Analysis

Continuous variables are presented as median with interquartile range [IQR] and categorical variables as frequency with percentage (%). Continuous and categorical variables were compared between groups using the Mann–Whitney U test and the chi-square or Fisher’s exact test, as appropriate. A time-to-event analysis was performed for treatment failure. Patients were followed from first positive ATI or ATA until treatment failure or the end of follow-up (May 2024). A receiver operating characteristic (ROC) analysis was performed to identify an ATA and ATI level threshold associated with treatment failure. Optimal thresholds were chosen using the Youden index. Quartile analyses of ATA and ATI levels were also performed, and quartiles were compared using the linear-by-linear association. Variables associated with treatment failure were identified by univariable and multivariable analysis logistic regression analysis using the Wald Backward selection method and included only statistically significant variables (*p*-value < 0.05) from the analysis. All analyses were performed using SPSS version 25.0 (SPSS, Chicago, IL, USA) and GraphPad Prism version 5.03 for Windows (GraphPad Software, San Diego, CA, USA).

## 3. Results

### 3.1. Study Population

The study population consisted of 134 patients with IBD [58 (43%) on adalimumab; 86 (64%) with CD] (Table 1).

The median age of diagnosis was 27 (IQR: 20–38) years, and 55% of the study population were male. There were no differences in the demographics and clinical data between patients treated with infliximab or adalimumab (Table 1). Patients were followed for a median of 38.3 (IQR: 26–83.9) months.

The first positive ADA was noted by reactive TDM in 78 (58.2%) patients and by proactive TDM in 56 (41.8%) patients (Table 1). The number of TDM tests per patient ranged from 1 to 9. The median time for detecting first positive ADA was 10.2 (IQR: 4.9–27.1) months. At the time the ADA were detected, the median infliximab and adalimumab concentrations were 0 (IQR: 0.0–3.7) μg/mL and 0 (IQR: 0.0–6.2) μg/mL, respectively. The median ATI and ATA levels were 8.3 (IQR: 5.7–29.1) U/mL and 3.4 (IQR: 4.9–14.0) U/mL, respectively.

### 3.2. Antibodies to Adalimumab

Patients with ATA and undetectable drug concentrations had higher median ATA levels compared to patients who still had detectable drug concentrations (11.9 [IQR: 4.3–56.0] vs. 3.8 [IQR: 2.9–5.1] U/mL, respectively, vs. *p* < 0.001). Patients with ATA which were first identified by proactive TDM had similar median ATA levels compared to patients with reactive TDM (5.1 [IQR: 3.7–11.8] vs. 4.8 [IQR: 2.9–21.0] U/mL, respectively, vs. *p* = 0.502). There were fewer patients with undetectable drug concentrations and ATA that were first identified by proactive TDM rather than reactive TDM (42% vs. 59%, respectively, vs. *p* = 0.270). Furthermore, there were fewer patients with undetectable drug concentrations and ATA identified by proactive TDM as compared to reactive TDM.

### 3.3. Antibodies to Infliximab

Patients with ATI and undetectable drug concentrations had higher median ATI levels compared to patients who still had detectable drug concentrations (21.3 [IQR: 8.8–60.7] vs. 6.2 [IQR: 5.2–7.8] U/mL, respectively, vs. *p* < 0.001). Patients with ATI who were first identified by proactive TDM had lower median ATI levels than patients who had undergone reactive TDM (7 [IQR: 5.4–8.6] vs. 22 [IQR: 7.6–58.9] U/mL, respectively, vs. *p* < 0.001). Moreover, there were fewer patients with undetectable drug concentrations and ATI identified by proactive compared to reactive TDM (29.7% vs. 74.3%, respectively, vs. *p* < 0.001).

### 3.4. Treatment Failure

**Figure 1:**
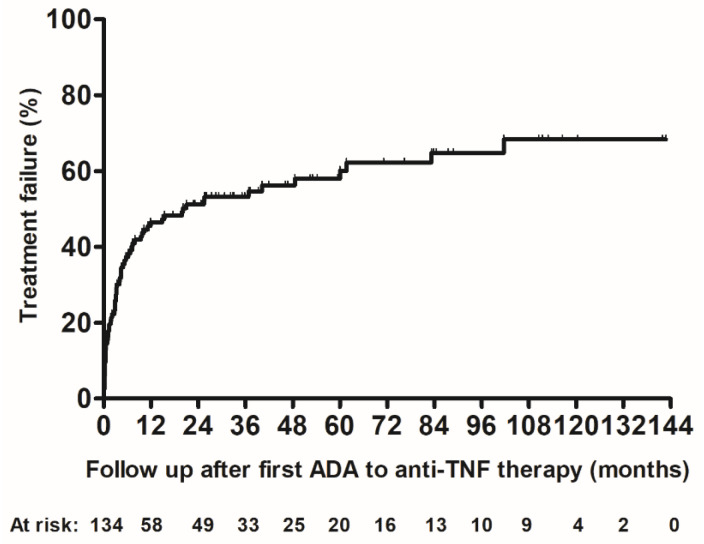
Kaplan–Meier cumulative probability curve of treatment failure in patients with IBD and anti-drug antibodies to anti-TNF therapy. ADA: anti-drug antibodies; TNF: tumor necrosis factor.

Overall, 62.7% of patients with ADA had treatment failure (Figure 1). Treatment failure was similar for patients treated with infliximab or adalimumab (63.2% vs. 62.0%, respectively; Log Rank, *p* = 0.728).

Patients with ADA and undetectable drug concentration had higher treatment failure compared to patients with detectable drug concentration (Log Rank, *p* = 0.025), although this did not remain statistically significant in multiple COX regression analysis (Table 2).

**Figure 2:**
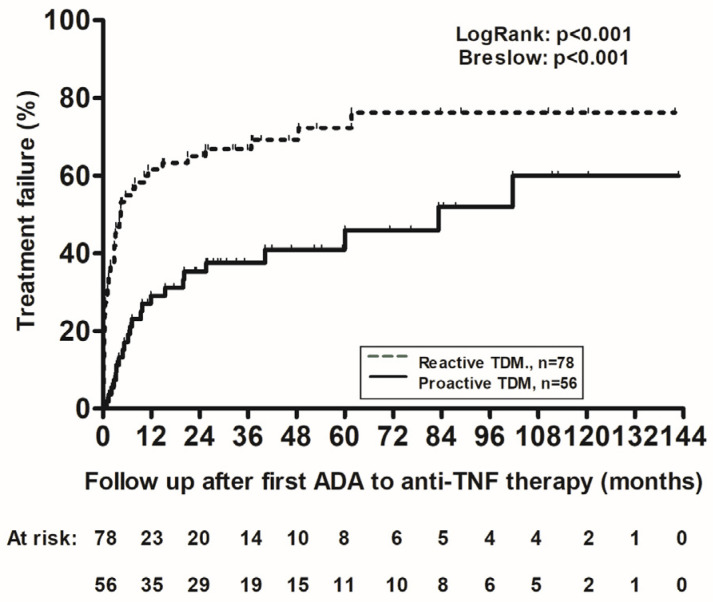
Kaplan–Meier cumulative probability curves of treatment failure in patients with IBD and anti-drug antibodies to anti-TNF therapy undergoing either reactive (dotted line) or proactive (solid line) TDM. ADA: anti-drug antibodies; TNF: tumor necrosis factor.

Multiple COX regression analysis identified higher ADA levels [hazard ratio (HR): 1.034, 95% confidence interval (CI): 1.024–1.045, *p* < 0.001)] and reactive TDM (HR: 2.398, 95%CI: 1.394–4.115, *p* = 0.002, Figure 2) to be associated with treatment failure.

### 3.5. Treatment Failure and Antibodies to Adalimumab or Infliximab

**Figure 3:**
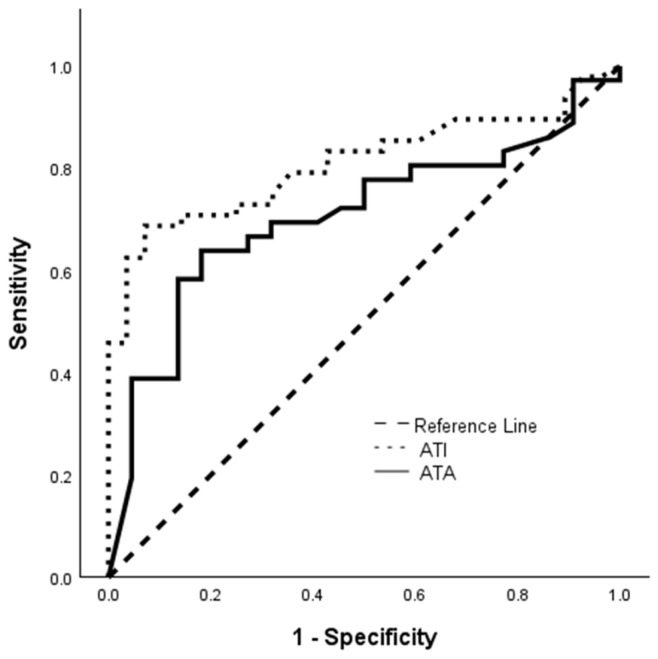
Receiver operating characteristic analysis of ATA and ATI levels distinguishing patients with or without treatment failure. ATA: antibodies to adalimumab; ATI: antibodies to infliximab.

**Figure 4:**
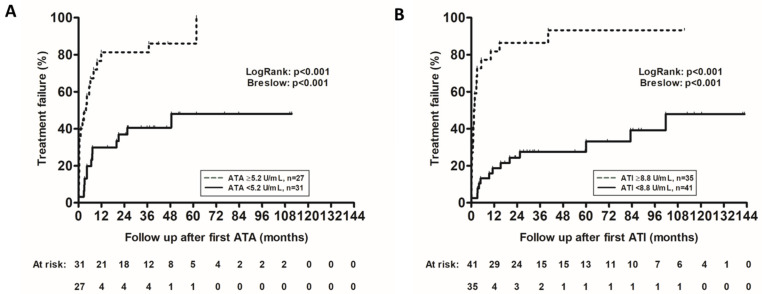
Kaplan–Meier cumulative probability curves of treatment failure in patients with IBD and ATA levels ≥ 5.2 U/mL (dotted line) vs. ATA levels < 5.2 U/mL (solid line) (**A**) or ATI levels ≥ 8.8 U/mL (dotted line) vs. ATI levels < 8.8 U/mL (solid line) (**B**). ATA: antibodies to adalimumab; ATI: antibodies to infliximab.

**Figure 5:**
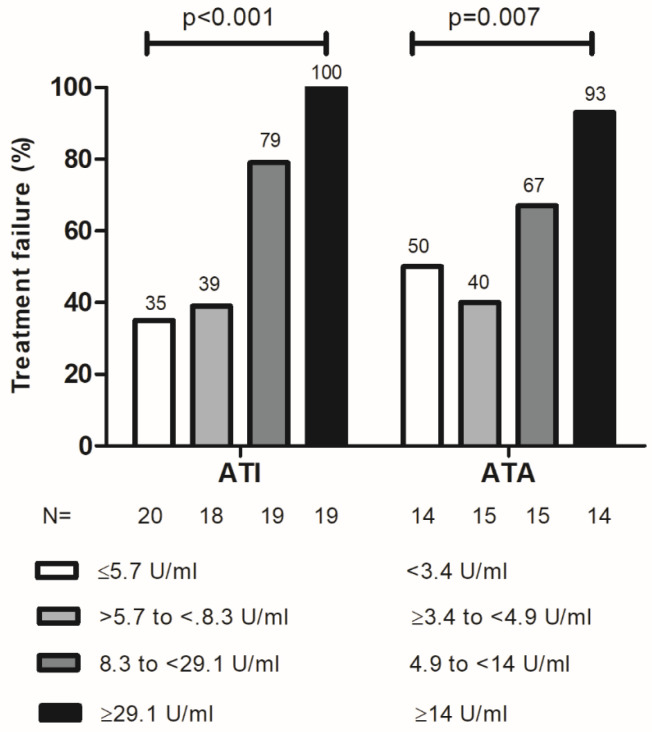
Quartile analysis of ATA and ATI levels associated with treatment failure. ATA: antibodies to adalimumab; ATI: antibodies to infliximab.

A ROC analysis identified an ATA titer threshold of 5.2 U/mL to distinguish patients with or without treatment failure (area under the ROC curve: 0.705; 95%CI: 0.569–0.841; *p* = 0.003; sensitivity: 64%; specificity: 82%, Figure 3). Patients with ATA ≥ 5.2 U/mL had a higher rate of treatment failure (HR: 4.227; 95%CI: 2.129–8.594; *p* < 0.001, Figure 4A). Quartile analysis of ATA levels associated with treatment failure is depicted in Figure 5.

A ROC analysis identified an ATI titer threshold of 8.8 U/mL to distinguish patients with or without treatment failure (area under the ROC curve: 0.809; 95%CI: 0.713–0.906; *p* < 0.001; sensitivity: 69%; specificity: 93%, Figure 3). Patients with ATI ≥ 8.8 U/mL had a higher rate of treatment failure (HR: 7.081; 95%CI: 3.696–13.564; *p* < 0.001, Figure 4B). A quartile analysis of ATI levels associated with treatment failure is depicted in Figure 5.

## 4. Discussion

Cumulative data suggest that immunogenicity to anti-TNF therapy can lead to unfavorable outcomes in IBD, though lower levels of ADA can be overcome and clinical benefit can be recaptured or maintained [9,10,11,12,13,14,15,16,17]. However, most of the data refer to infliximab, and there is very limited data regarding adalimumab.

We demonstrated that immunogenicity to anti-TNF therapy can lead to treatment failure in around 63% of patients with ADA, especially in patients with higher ADA levels. Furthermore, our study identified a cut-off of 5.2 U/mL for ATA that distinguished patients with or without treatment failure. This is the first to identify an ATA level associated with clinical outcomes. We also identified a cut-off of 8.8 U/mL for ATI that distinguished patients with or without treatment failure. This ATI threshold is similar to one reported in a previous study, which demonstrated that in patients with loss of response to infliximab and ATI < 9.1 U/mL, dose optimization is a valid therapeutic option [11]. Moreover, a study by Battat and colleagues, including patients with IBD and immunogenicity to infliximab, showed that dose escalation eliminated an initial ATI level of ≤ 8.6 U/mL with increased efficacy [14].

We also demonstrated that immunogenicity to anti-TNF therapy leads to treatment failure regardless of whether the drug is detectable when ADA are identified or whether the drug is adalimumab or infliximab. This is in line with a previous study showing that ATI can negatively influence the efficacy of infliximab even in low concentrations and in the presence of an adequate trough drug concentration [11]. Our study is also the first to show that reactive, compared to proactive, TDM is independently associated with higher treatment failure in patients with ADA. This was previously shown for both infliximab and adalimumab in a larger population of patients with IBD, not all with ADA [22]. It seems that reactive TDM is likely implemented too late in many patients, as high level ADA may have already developed.

Strengths of this study include the rather large sample size for such a unique population, the representation of real-life clinical practice, and the utilization of a drug-tolerant assay that can allow the detection of ADA even in the presence of drug concentration [18]. However, this study is limited by its retrospective design with an inherent potential for selection bias and the fact that causality cannot be established. Moreover, due to the retrospective design of the study, it was not feasible to control/account for the type of treatment modifications used to overcome immunogenicity, which are often heterogeneous. Additionally, the results may not be generalizable, as they represent clinical practice in a single large academic medical center. We would also like to highlight that the results of this study apply to patients who develop ADA rather than to all patients initiating anti-TNF therapy.

In conclusion, this large retrospective cohort study showed that most patients with immunogenicity to anti-TNF therapy experience treatment failure, particularly those with higher levels of ADA identified by reactive TDM. Furthermore, we identified ATI and ATA level thresholds of 8.8 and 5.2 U/mL, respectively, associated with treatment failure. Large prospective studies are needed to confirm these results.

## Figures and Tables

**Table 1:** Patient demographic and clinical characteristics.

Patient Characteristics	Total Cohort (N = 134)	Adalimumab (N = 58)	Infliximab (N = 76)	*p*-Value
Male, (%)	74 (55)	34 (59)	40 (53)	0.599
Age at diagnosis, median (IQR), years	27 (20–38)	26.5 (20–38)	29 (21–40)	0.236
CD, (%) ^#^	86 (64)	37 (64)	49 (64)	0.857
UC extension, (%)				0.736
E1 (proctitis)	5/47 (10)	2/21 (10)	3/26 (12)	
E2 (left-sided colitis)	13/47 (28)	7/21 (33)	6/26 (23)	
E3 (pancolitis)	29/47 (62)	12/21 (57)	17/26 (65)	
CD location, (%)				0.431
L1 (ileal)	32/86 (37)	17/37 (46)	15/49 (31)	
L2 (colonic)	17/86 (20)	6/37 (16)	11/49 (22)	
L3 (ileocolonic)	37/86 (43)	14/37 (38)	23/49 (47)	
CD behavior, (%)				0.401
B1 (non-stricturing, non-penetrating)	36/86 (42)	15/37 (41)	21/49 (43)	
B2 (stricturing)	22/86 (25)	12/37 (32)	10/49 (20)	
B3 (penetrating)	28/86 (33)	10/37 (27)	18/49 (37)	
Perianal fistulizing disease, (%)	26/86 (30)	9/37 (24)	17/49 (35)	0.349
Prior ileocolonic resection, (%)	27/86 (31)	16/37 (43)	11/49 (22)	0.060
Smoking ever, (%)	34 (25)	15 (26)	19 (25)	1.000
Prior biological therapy, (%)	45 (34)	21 (36)	24 (32)	0.585
IMM ever, (%)	72 (54)	33 (57)	39 (51)	0.601
Albumin *, median (IQR), g/dL	4.5 (4.1–4.7)	4.4 (4.1–4.7)	4.5 (4.1–4.8)	0.874
Concomitant IMM *, (%)	36 (13)	28 (13)	8 (13)	1.000
BMI *, median, IQR, kg/m^2^	26.8 (23.8–30.9)	25.7 (23.9–31.1)	26.9 (23.7–30.9)	0.759
CRP *, median (IQR), mg/L	2.7 (1.1–7.4)	1.9 (0.9–4.5)	3.5 (1.1–10.8)	0.073
Reactive TDM, (%)	78 (58)	39 (67)	39 (51)	0.078

^#^ One patient had a pouch. * At first TDM with immunogenicity. CD: Crohn’s disease; UC: ulcerative colitis; IMM: immunomodulators; IQR: interquartile range; TDM: therapeutic drug monitoring; CRP: C-reactive protein. BMI: body mass index.

**Table 2:** Variables associated with time to treatment failure to anti-TNF therapy.

	Univariate Analysis			Multivariate Analysis		
Variables	HR	95%CI	*p*-Value	HR	95%CI	*p*-Value
Male	1.3	0.9–2.1	0.158			
Age at diagnosis	1.01	0.99–1.02	0.417			
CD	1.2	0.8–1.9	0.437			
UC extension	0.9	0.6–1.5	0.744			
CD location	0.9	0.6–1.2	0.344			
Type of anti-TNF therapy	0.9	0.6–1.4	0.752			
CD behavior	1.1	0.8–1.6	0.420			
Perianal fistulizing disease	1.2	0.7–2.1	0.522			
Prior ileocolonic resection	1.1	0.6–1.9	0.754			
Smoking ever	1.7	1.1–2.7	0.020			
Albumin *	0.9	0.5–1.7	0.675			
Prior biological therapy	0.8	0.5–1.3	0.385			
Concomitant IMM *	0.5	0.2–1.3	0.156			
BMI *	0.9	0.9–1.1	0.433			
Drug concentration *	0.9	0.8–0.9	<0.001			
CRP *	1.01	1.01–1.02	0.005			
ADA with undetectable drug concentration *	1.6	1.1–2.5	0.025			
ADA level *	1.04	1.03–1.04	<0.001	1.03	1.02–1.04	<0.001
Reactive TDM	2.9	1.8–4.7	<0.001	2.4	1.4–4.1	0.002

* At first TDM with immunogenicity. CD: Crohn’s disease; UC: ulcerative colitis; IMM: immunomodulator; CI: confidence interval; TDM: therapeutic drug monitoring; CRP: C-reactive protein. ADA: anti-drug antibodies; BMI: body mass index; TNF: tumor necrosis factor.

## Data Availability

The data that support the findings of this study are available upon reasonable request.
